# Adapted version of the Pubertal Development Scale for use in Brazil

**DOI:** 10.11606/s1518-8787.2019053000915

**Published:** 2019-08-12

**Authors:** Sabine Pompéia, Gislaine de Almeida Valverde Zanini, Rafaella Sales de Freitas, Luanna Maristella Cabanal Inacio, Flávia Calanca da Silva, Giovana Ribeiro de Souza, Maria Sylvia de Souza Vitalle, Sheila Rejane Niskier, Hugo Cogo-Moreira

**Affiliations:** I Universidade Federal de São Paulo. Departamento de Psicobiologia. São Paulo, SP, Brasil; II Universidade Federal de São Paulo. Programa de Pós-Graduação em Psicobiologia. São Paulo, SP, Brasil; III Universidade Federal de São Paulo. Programa de Pós-Graduação em Saúde Coletiva. São Paulo, SP, Brasil; IV Universidade Federal de São Paulo. Setor de Medicina do Adolescente. Departamento de Pediatria. São Paulo, SP, Brasil; V Universidade Federal de São Paulo. Departamento de Psiquiatria. São Paulo, SP, Brasil

**Keywords:** Adolescent Development, Puberty, Surveys and Questionnaires, Translations, Reproducibility of Results

## Abstract

**OBJECTIVE:**

To determine whether scores in an adapted version of the self-assessment Pubertal Development Scale into Portuguese match those from the gold standard in pubertal development (Tanner scale).

**METHODS:**

This was a cross-sectional study with a convenience sample of 133 children and adolescents aged nine to 17 years (59 males; mean age of 13 years and six months, with standard deviation = 25 months). Youngsters completed the Pubertal Development Scale and were then examined by specialists in adolescent medicine.

**RESULTS:**

Exact absolute agreement of pubertal stages were modest, but significant associations between measures (correlation; intra-class correlation coefficients of consistency) showed that the Pubertal Development Scale adequately measures changes that map onto pubertal development determined by physical examination, on par with international publications. Furthermore, scores obtained from each Pubertal Development Scale question reflected adequate gonadal and adrenal events assessed by clinical ratings, mostly with medium/high effect sizes. Latent factors obtained from scores on all Pubertal Development Scale questions had excellent fit indices in Confirmatory Factor Analyses and correlated with Tanner staging.

**CONCLUSIONS:**

We conclude that self-assessment of body changes by youngsters using the Portuguese version of the Pubertal Development Scale is useful when estimates of pubertal progression are sufficient, and exact agreement with clinical staging is not necessary. The Pubertal Development Scale is, therefore, a reliable instrument for use in large-scale studies in Brazil that aim at investigating adolescent health related to pubertal developmental. The translated version and scoring systems are provided.

## INTRODUCTION

Puberty involves a set of neuroendocrine changes that occur during the transition from childhood to sexual maturity^1^^,^^2^. Age is not a good predictor of pubertal development because the timing of puberty and individual’s puberty relative to that of others of the same age and sex and also the progression speed (tempo) to full sexual maturity vary widely and depend on genetic, ethnic, nutritional, and psychosocial factors^2–4^. Because of this variability, adequate assessment of pubertal onset and progression is crucial to detect disorders that may affect this process^2^^,^^5^. This is also important in studies that aim to better understand adolescence and how it relates to a variety of biopsychosocial factors.

The gold standard in pubertal development rating is the Tanner scale (e.g., Tanner and Marshal)^6^^,^^7^, also known as Tanner stages and Tanner rating. This method classifies puberty into five progressive stages^6^^,^^7^, considering changes that occur independently^2^ in: a) size and shape of the breasts in girls and genitals in boys, which reflect mainly activation of the hypothalamic-pituitary-gonadal axis^1^; and b) the distribution and characteristics of pubic hair in both sexes, which reflect increased output of steroids due to the expansion of the adrenal *zona reticularis*^8^.

The Tanner scale requires physical examinations conducted by extensively trained clinicians^2^. Ratings using this method are therefore not always possible due to the high costs of hiring these professionals and providing adequate settings for examinations, especially in studies with large samples^2^. To circumvent these limitations, an alternative method for pubertal staging was proposed: the Pubertal Development Scale (PDS)^9^^,^^10^. The PDS is a self-assessment scale composed of five questions that enquire about gonadal, adrenal and growth factors that alter the body during puberty^1,3,8,11–14^, which is therefore multidimensional in terms of assessing neuroendocrine changes in this phase of life. This scale does not have illustrations of pubertal stages; it does not mention genitalia, nor involve been seen naked or palpated. Thus, this scale is extensively used in the literature because it is less embarrassing for youngster, is cheaper and easier to administer than Tanner ratings, and can be applied in a variety of settings (e.g., in schools or in mail or online studies) and populations^2^. Furthermore, PDS scores capture genetic and nonshared environmental factors that influence pubertal development^15^ and correlate both with bone mineral density/mineral content^16^ and with gonadal and adrenal hormone concentrations^17^^,^^18^, in some cases even more so than clinical staging^17^.

Petersen et al.^9^ and Carskadon and Acebo^10^ claimed that the PDS has adequate validity, but only the latter compared PDS scores to Tanner staging, and did so only with a small group of participants. In fact, very few studies have compared PDS scores to clinical ratings ^2^, which is surprising considering the popularity of this self-assessment scale. Internal consistency reliability (Cronbach’s alpha) of the PDS range from questionable to acceptable^9^^,^^10^^,^^19^.

The aims of the study proposed here were to: a) adapt the PDS for use in Brazil; and b) determine the extent to which self-assessment with this translated scale corresponded to clinical Tanner staging rated by physicians trained in adolescent medicine (validity criterion) using a variety of statistical approaches, including Structural Equation Modeling.

## METHODS

### Participants

Consecutive Portuguese speaking patients aged nine to 17 years who attended medical centers that treat adolescents at the Hospital São Paulo, in Brazil, for four months. The only exclusion criterion was reporting difficulties in reading and writing. Patients with health conditions influencing pubertal development were not excluded because our objective was to study the relationship between clinical Tanner staging and self-assessment of pubertal development, rather than to characterize puberty onset, timing, or tempo.

### Procedure

Firstly, the scale was translated into Portuguese (see Adaptation process below). We then tested the adequacy of the adapted version in a cross-sectional study approved by the Ethics Committee at the institution in which the study took place (#2.001.042). Consecutive patients were approached in waiting rooms before their consultations and received explanations about the study. Those who agreed to take part in the study provided written informed consent or assent. Guardians filled out a demographic questionnaire that also enquired about age of menarche if applicable. Youngsters were asked to complete the PDS and other behavioral questionnaires that will not be addressed here. Participants then underwent their consultations, during which physicians rated their pubertal stage on the Tanner scale. Physicians were blind to patients’ self-ratings of pubertal development.

### Adaptation process of the PDS into Portuguese (Box)

The original PDS proposed by Carskadon and Acebo^10^ was translated into Portuguese by a native speaker of both English and Portuguese, and back-translated by a person with similar language skills. Both translated and back-translated versions were then analyzed by three specialists in adolescent medicine and three researchers specialized in cognition. They determined the adequacy of the translated version and proposed slight alterations to improve the understanding of the scale: 1) The Likert rating “seems complete” was found to be unclear, so details pertaining each question were added to indicate maximum development had been reached (e.g., for growth spurt, the following was added: “I am not growing so fast any longer”); 2) A term in Portuguese for growth spurt (*estirão de crescimento*) exists, so it was added in brackets after “rapid growth in height”; 3) For the word “breast” we also included various alternative terms that are used in Brazil (*peitos, seios, mamas*). The scale in Portuguese, the instructions for completion and the scoring system can be found in Box 1.

**Box:** The Pubertal Development Scale (PDS) in the translated Portuguese versions: the instructions for completion (A), the Portuguese scale (B) and information needed for scoring (C and D).

A. Instructions for completion (in Portuguese)						
“*As próximas 5 perguntas são sobre as mudanças que podem estar acontecendo com o seu corpo. Essas mudanças normalmente acontecem de forma diferente em cada pessoa e idade. Por favor faça um ‘x’ na alternativa que melhor reflete as mudanças que você está percebendo no seu corpo. Se não entender uma pergunta ou não souber a resposta, marque um x em ‘não sei’ ou pergunte se houver alguém aplicando a escala*”						
B. The Pubertal Development Scale (in Portuguese)						
*Pergunta*		*Ainda não começou*	*Parece que começou*	*Começou com certeza*	*Parece completo*	*Não sei*
For males (*Para garotos*)						
*Você diria que seu crescimento rápido em altura (estirão de crescimento)*					*“Não estou crescendo mais tão rápido”*	
*Você diria que o crescimento dos pelos no seu corpo (como embaixo dos braços, sem considerar cabelos na cabeça)*					*“Parou de aumentar”*	
*Você notou mudanças na pele, especialmente espinhas?*					*“Não está mais aumentando”*	
*Você notou engrossamento da voz?*					*“Não está mais engrossando”*	
*Começou a crescer pelos no seu rosto?*					*“Não está mais aumentando”*	
For females (*Para garotas*)						
*Você diria que seu crescimento rápido em altura (estirão de crescimento)*					*“Não estou crescendo mais tão rápido”*	
*Você diria que o crescimento dos pelos no seu corpo (pelos como embaixo dos braços, sem considerar cabelos na cabeça)*					*“Parou de aumentar”*	
*Você notou mudanças na pele, especialmente espinhas?*					*“Não está mais aumentando”*	
*Você notou que seus peitos (seios ou mamas) começaram a crescer?*					*“Não estão mais crescendo”*	
	
*Você já menstruou?*		*Não ( ) Sim ( ) → Que idade tinha quando menstruou pela 1ª vez?___ Lembra mês? ___ Ano?___*_				

C. Scoring^10,19^						
For all questions except menarchal status (“*já menstruou*”): “*ainda não começou*” = 1 point; “*parece que começou*” = 2 points; “*começou com certeza*” = 3 points; “*parece completo*” = 4 points; “*não sei*” = 0 (missing values). For menarche: “no” = 1 point; “yes” = 4 points. We suggest that one “I don’t know” or one missing answer be substituted for the mean points on the other 4 items.						
D. Computation of Pubertal Category Score (PCS) based on Carskadon and Acebo^10^ except for pre-pubertal scores for girls^19^						
Correspondence to Tanner staging	Males (add points relative to voice changes, facial and body hair growth as indicated in C-scoring)			Females (add points relative to body hair and breast growth and consider menarche as indicated in C-scoring)		
1 (pre-pubertal)	3 points			2 points (with no menarche)		
2 (early-pubertal)	4–5 points (with no 3-point answers)			3 points (with no menarche)		
3 (mid-pubertal)	6–8 points (with no 4-point answers)			4–8 points (with no menarche)		
4 (late-pubertal)	9–11 points			1–7 points (with menarche)		
5 (post-pubertal)	12 points			8 points (with menarche)		

### Measures

- Clinical Tanner staging^6^^,^^7^ by physical examination: participants’ pubertal staging (stage 1 to 5) was rated by development of genitals in boys, breasts in girls, and pubic hair in both sexes. Palpation of testes and breasts was performed in early genital stages^2^. When in doubt between two adjacent stages, clinicians were instructed to rate patients with the mean stage^20^. In the facilities where male and female patients were examined, ratings were conducted by specialists in adolescent health and one resident at the end of her training in the field. Until the middle of the study, all ratings were checked by a single expert. As no inconsistencies were found, we did not assess inter-rater variability.
- The participants’ self-reported development using the translated and adapted version of the Pubertal Development Scale (PDS)^10^: the PDS is a self-assessment instrument (see Box) composed of five questions pertaining to growth spurt, body hair, and changes in skin for both sexes, rated on 5-point scales: “not yet started” (scored as one point), “barely started” (two points)”, “definitely started” (three points)”, “seems complete” (four points), “I don’t know” (treated as missing values). On a similar scale, males also rate their development regarding changes in voice and facial hair growth, while females rate breast growth. Females also state whether they have begun to menstruate (scored 1 for “no” and 4 for “yes”). Note that many studies cite publications other than Carskadon and Acebo^10^ as the origin of the scale and scoring system, but these are unpublished manuscripts – except that the study by Petersen et al.^9^ was published, but their research does not include the scale, which can be found in Carskadon and Acebo’s^10^ article. We used three scoring systems^10^^,^^19^ (Box): a) PDS1 (the mean score of all five questions); b) PDS2 (sum of scores obtained from three sex-specific questions: voice changes and body and facial hair growth in males; menarche, body hair and breast growth in females); and c) Puberty Category Score (PCS)^19^: scores from the same questions used to calculate PDS2 converted into stages akin to Tanner stages. However, in the latter case PCS scoring for girls was based on Chan et al.^19^ and differs from the one described in Carskadon and Acebo^10^ for stage 1 (3 points = stage 1), because in this publication^10^ stage 1 is undistinguishable from stage 2 (3 points with no menarche = stage 2).

### Statistical Analysis

Correspondence between clinical pubertal ratings (each of the Tanner stages for breasts, male genitalia, male and female pubic hair) and self-assessment (three types of PDS scores), separately for each sex, were analyzed with: a) Spearman’s rank rho correlations; b) Kendall’s tau correlation coefficients, which measures the strength of cross tabulations; c) intra-class correlations coefficients (ICC), with 95% confidence intervals, using SPSS software version 23 and considering independent evaluators, mixed two-way models^21^ – consistency ICC were calculated when Tanner ratings were compared to continuous scores (PDS1 and PDS2), while absolute agreement ICC was only obtained when Tanner ratings were compared to PCS, since only in this case measures are in the same ordinal scale; and d) weighted Kappa values (WK)^22^ with 95% confidence interval (95%CI)^22^, comparing only the PCS with Tanner staging (expressed in the same scale), using the online tool vassarstats.net.

The ICC values below 0.50 are considered of low reliability, those between 0.50–0.75 are regarded as of moderate reliability, those between 0.75–0.90 are of good reliability, and those between 0.90–1.00 are considered excellent^21^. The WK is an agreement statistic for ordinal categories and the proportion of weighted agreement is corrected for chance. It involves giving high weights to agreement and responses that differ by one category, lower weights if they differ by two and three categories, while no weight is given if they differ by more categories. The benchmarks for WK values are: 0.00–0.20 = slight agreement; 0.21–0.40 = fair agreement; 0.41–0.60 = moderate agreement; 0.61–0.80 = substantial agreement; > 0.8 = almost or perfect agreement.

We also ran general linear models (GLM) separately for each sex to determine the proportion of variance explained (multiple R^2^) in the three PDS scores (dependent continuous variables) by clinical staging (regarded as a continuous predictor) on the three types of PDS scores in different models. Other GLM were also used to determine the extent to which the answers to each PDS question (used as factors with each question as a level, each of which with scores ranging from one to four) were associated with pubic hair and breasts or genital Tanner staging depending on the sex of the participant (continuous predictor) in different models, separately for males and females. R^2^ were used to determine effect sizes: values between 0.13 and 0.25 are considered medium, and those over 0.26, large^23^.

Structural equation modeling was used to determine the adequacy of a latent factor obtained from raw scores of all the PDS questions. To do so, we carried out confirmatory factor analyses (CFA) on scores of the scale using Mplus version 8.0^24^. The aims of these analyses were to: 1) test PDS measurement model fit; 2) evaluate the magnitude of correlation between each PDS item and an underlying pubertal development factor. To evaluate the goodness of fit of the statistical model we considered^25^: chi-square, confirmatory fit index (CFI), Tucker-Lewis index (TLI), Root Mean Square Error Approximation (RMSEA), and Standardized Root Mean Square Residual (SRMR). The following cutoff criteria were used to determine a well-fitted model^25^: a) non statistically significant chi-square (p > 0.05); b) RMSEA near or less than 0.06 and a non-significant close fit (Cfit - p > 0.05), a statistical test of closeness of model fit using RMSEA; and c) CFI and TLI near or greater than 0.95 and SRMR less than 0.08.

The PDS internal consistency has been assessed mainly using Cronbach’s alpha in the literature, with values varying from questionable to substantial^10^^,^^11^^,^^19^. This is so in the original version in English^9^^,^^10^ and the PDS versions translated into other languages (see Discussion). However, this classical index of internal consistency has many shortcomings^25^. Assumptions must be met before estimating alpha, which is only a good estimate of reliability for congeneric measures if: a) measures are true score equivalent and errors of measurement are uncorrelated; or b) errors are uncorrelated and the components load uniformly highly on the common latent dimensions^25^.

These assumptions were never considered when calculating alphas using PDS scores in prior investigations. Consequently, the previous reported alphas might be under- or over-estimated, so evidence regarding PDS internal consistency are still lacking. To overcome the disadvantages of Cronbach’s alpha, scale reliability for male and female models was estimated via factor loadings of CFA^26–28^ in models in which measurement errors are uncorrelated (in the case of the model with data from males), and based on Raykov^27^ for the model with female data (due to the inclusion of a correlation between a pair of residual variances; see the Results section). Lastly, we used Pearson correlation to evaluate the degree of association between the pubertal latent variable with the two clinical assessments regarding body hair growth and breast or male genitals.

## RESULTS

Of the 187 youngsters approached in the waiting rooms, 148 (84 females) accepted to take part in the study. Here we describe results on the nine to 17 year-olds from whom both Tanner staging and PDS were available: 133 individuals (mean age 13 years and six months; SD = 25 months) of whom 59 were males (aged 13 years and eight months; SD = 24 months) and 74, females (aged 13 years and five months; SD = 27 months). We found no discrepancies between reports of guardians’ and participants’ menarche status and age of menarche.

Intermediate ratings between two stages were used by clinicians for only 4.5% of the ratings. For the absolute agreement analyses (ICC and WK), these mean ratings were rounded down because the next stage had not yet been reached. Male participants in each of the Tanner genital stages were: eight (stage 1); nine (stage 2); 11 (stage 3); nine (stage 4); 22 (stage 5). For females breast stages: two (stage 1); five (stage 2); six (stage 3); 21 (stage 4); 40 (stage 5).

Correlations between the clinical and PDS scores are in Table 1. Consistency between physical ratings and PDS scores (ICC) was mostly moderate to good, while ICC absolute agreement and WK results for the PCS were modest (Table 2). Findings obtained from the GLM investigating the extent to which Tanner staging explained the variance in the three PDS scores reached high effects sizes (Table 3). Tanner ratings also significantly explained PDS ratings when answers to individual questions were levels of a PDS factor (Table 3).

**Table 1:** Spearman and Kendall tau significant (p < 0.0001) correlations between pubertal staging using Tanner ratings by physical examination and scores on the self-assessed pubertal development using the Pubertal Development Scale (PDS), per sex.

Statistics	PDS score	Males (n = 59)		Females (n = 74)	
	
		Genitals	Pubic hair	Breasts	Pubic hair
Spearman r	PDS1	0.77	0.78	0.74	0.65
	PDS2	0.75	0.72	0.52	0.41
	PCS	0.72	0.67	0.74	0.62
Kendall tau	PDS1	0.63	0.64	0.64	0.56
	PDS2	0.60	0.60	0.46	0.37
	PCS	0.61	0.58	0.70	0.58

PDS1 = mean score of answers to all five PDS questions; PDS2 = sum of score of answers to three PDS questions; PCS (Pubertal Category Score) = pubertal staging equivalent to the Tanner scale obtained from the PDS2 scoring system (see Box for details).

**Table 2:** Intra-class correlation coefficients (ICC) and weighted Kappa values with 95% confidence intervals (95%CI) comparing pubertal staging using Tanner ratings by physical examination and scores on the self-assessed pubertal development using the Pubertal Development Scale (PDS), per sex.

Type of clinical Tanner staging	PDS score types	Comparison statistic	Males (n = 59)	Females (n = 74)
Pubic hair	PDS1	ICC (consistency)	0.70 (95%CI 0.50–0.82)	0.77 (95%CI 0.64–0.85)
	PDS2	ICC (consistency)	0.84 (95%CI 0.74–0.90)	0.78 (95%CI 0.65–0.86)
	PCS	ICC (agreement)	0.66 (95%CI 0.28–0.82)	0.69 (95%CI 0.31–0.84)
		Weighted Kappa	0.32 (95%CI 0.21–0.43)	0.25 (95%CI 0.11–0.39)
				
Male genitals/	PDS1	ICC (consistency)	0.73 (95%CI 0.54–0.83)	0.83 (95%CI 0.73–0.90)
Female breasts	PDS2	ICC (consistency)	0.82 (95%CI 0.70–0.90)	0.81 (95%CI 0.70–0.89)
	PCS	ICC (agreement)	0.68 (95%CI 0.30–0.84)	0.80 (95%CI 0.44–0.90)
		Weighted Kappa	0.30 (95%CI 0.20–0.40)	0.42 (95%CI 0.28–0.56)

PDS1 = mean score of answers to all 5 PDS questions; PDS2 = sum of score of answers to three PDS questions; PCS (Pubertal Category Score) = pubertal staging equivalent to the Tanner scale obtained from the PDS2 scoring system (see Box for details). Absolute agreement was only tested when comparing ICC and Weighted Kappa of Tanner ratings and PCS as only these are rated on a similar scale.

ICC rules of thumb: < 0.50 = low reliability; 0.50–0.75 = moderate reliability; 0.75–0.90 = good reliability; 0.90–1.00 = excellent reliability; Weighted Kappa rules of thumb: 0.00–0.20= slight agreement; 0.21–0.40 = fair agreement; 0.41–0.60 = moderate agreement; 0.61–0.80 = substantial agreement; > 0.8 = almost perfect agreement.

**Table 3:** Results of general linear models per sex in which Tanner staging by physical examination were the continuous predictors, and Pubertal Development Scale (PDS) scores were entered as dependent continuous variables (either alone or as a factor with answers to individual questions as levels of a PDS factor).

Variable	PDS metrics	Tanner staging	R^2^	F^a^	p
Males					

Score	PDS1	Pubic hair	0.64	99.41	< 0.0001
		Genitalia	0.60	86.40	< 0.0001
	PDS2	Pubic hair	0.54	66.22	< 0.0001
		Genitalia	0.54	67.75	< 0.0001
	PCS	Pubic hair	0.40	51.82	< 0.0001
		Genitalia	0.45	55.87	< 0.0001
Questions as levels of a PDS factor	Growth spurt		0.31		
	Body hair growth		0.53		
	Skin changes	Pubic hair	0.31	97.00	< 0.0001
	Voice changes		0.47		
	Facial hair growth		0.20		
	Growth spurt		0.34		
	Body hair growth		0.44		
	Skin changes	Genitalia	0.23	86.02	< 0.0001
	Voice changes		0.48		
	Facial hair growth		0.24		

Females					

Score	PDS1	Pubic hair	0.57	95.91	< 0.0001
		Breasts	0.68	150.37	< 0.0001
	PDS2	Pubic hair	0.31	33.11	< 0.0001
		Breasts	0.39	42.58	< 0.0001
	PCS	Pubic hair	0.51	74.03	< 0.0001
		Breasts	0.68	152.18	< 0.0001
Questions as levels of a PDS factor	Growth spurt		0.19		
	Body hair growth	Pubic hair	0.46	4.36^a^	< 0.006
	Skin changes		0.21		
	Breast growth		0.06		
	Growth spurt		0.21		
	Body hair growth	Genitalia	0.40	56.68	< 0.0001
	Skin changes		0.20		
	Breast growth		0.16		
	Menarche^b^	Pubic hair	0.52	77.10	< 0.0001
	Menarche^b^	Genitalia	0.65	133.60	< 0.0001

Multiple R^2^ are the proportion of variance explained in the PDS by the clinical ratings (R^2^ between 0.13 and 0.25 = medium effect size; R^2^ > 0.26 = large effect size)^23^. PDS1 = mean score of answers to all five PDS questions; PDS2 = sum of score of answers to three PDS questions considered in the PCS (Pubertal Category Score); PCS = pubertal staging equivalent to the Tanner scale obtained from the PDS2 scoring system). See Box for scoring systems.

Score per question involved combining all continuous scores as different levels in a PDS factor; Degrees of freedom: males 1,57; females 1,72.

^a^ interaction of clinical staging and the factor questions (degrees of freedom 3,216).

^b^ a separate model was run for menarche because it is answered with a dichotomous rating (yes or no).

**Figure 1:**
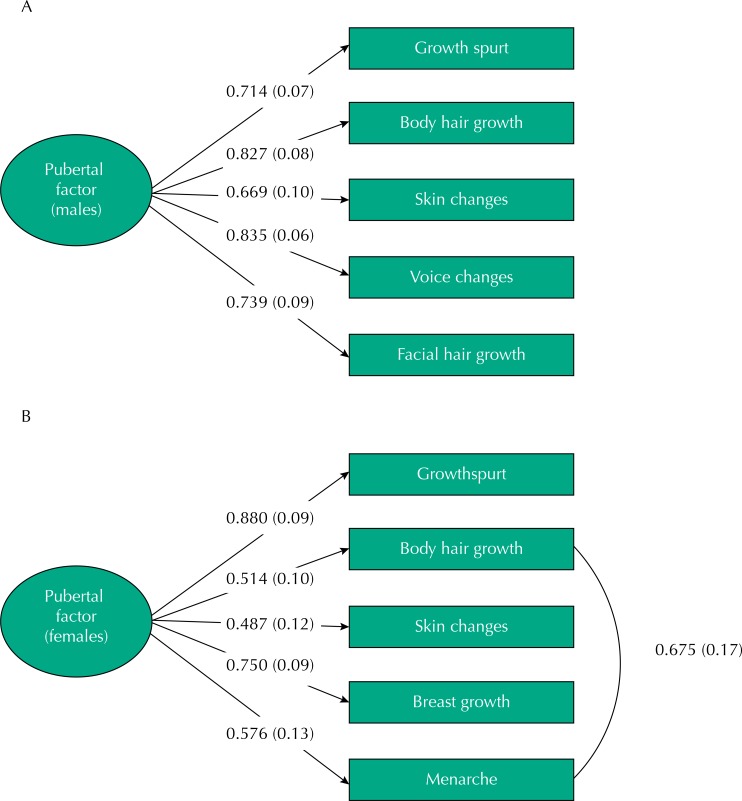
Confirmatory factor analysis of the Pubertal Development Scale (PDS) scores for the model obtained from data from males (A) and females (B), including factor loadings on the pubertal development factor (represented as the oval) and their respectively standard errors in brackets.

Note: Fit indices for males: χ_(5)_ = 3.393, p = 0.6397; RMSEA (root mean square error approximation) < 0.001 and Cfit (Close fit) = 0.714, CFI (Confirmatory Fit Index) = 1.000 and TLI (Tucker-Lewis index) = 1.013.

The CFA for males showed excellent fit indices (Figure 1-A). A similar model was run for females, but it did not fit the data well (χ_(5)_ = 16.741, p = 0.0050; RMSEA = 0.167 and Cfit = 0.015, CFI = 0.924 and TLI = 0.849), possibly because menarche is rated on a dichotomous scale and is less sensitive to the progressive changes during pubertal development. Because the PDS is multidimensional by reflecting both gonadal and adrenal changes assessed in the Tanner scale, we correlated residuals variance to menarche and growth of body hair, the two questions that best associated with gonadal and adrenal clinical ratings in the GLM, respectively. With this adjustment, the model for female showed excellent fit indices (Figure 1-B). Scale reliability based on CFA results were 0.87 (males) and 0.71 (females). Pearson correlations of the latent PDS factor and Tanner staging were significant (p < 0.001): males r = 0.786 (pubic hair) and r = 0.726 (genital development); females r = 0.541 (pubic hair) and r = 0.738 (breast development).

For females, the curved line represents an adjustment to the model, that is, a correlation of residual variances of menarche and growth of body hair (see Results section for details). Fit indices were: χ_(4)_ = 4.804, p = 0.3080; RMSEA = 0.049 and Cfit = 0.413; CFI = 0.995 and TLI = 0.987.

## DISCUSSION

Overall, we found that the PDS is a self-assessment instrument that captures changes in pubertal development assessed by experienced clinicians. PDS raw scores (especially PDS1), and Tanner stages correlated well. They were higher than those found for females in the study by Brooks-Gunn et al.^29^ and similar to values from Schmitz et al.^16^ The PDS1 score was also the one best associated with clinical staging in GLM. Over 60% of the PDS variance for males and over 57% for females was explained by Tanner ratings (high effect sizes). The three PDS scores had mostly similar ICC for consistency (moderate to good). Good to excellent values were obtained comparing PDS2 score for females regarding pubic Tanner staging, and when comparing PCS scores and the genital Tanner scale in males. Hence, all scoring methods have merits. Nevertheless, the PDS1 includes more changes related to puberty and might be a better estimate of changes in youngsters’ bodies.

Regarding absolute agreement between self-rated PCS score and clinical staging, results were less positive. Absolute agreement ICC and weighted Kappa values were mostly low to good and slight to fair, respectively, with wide ranges of confidence intervals, suggesting that this scoring system does not map well onto Tanner stages, corroborating findings of Schmitz et al.^16^ and Shirtcliff et al.^17^ This is not surprising considering that the PDS measures pubertal events that are not considered by Tanner ratings. Also, clinical pubertal rating on the Tanner scale entails comparing adolescents among each other and with photographs that reflect pubertal stages regarding specific body characteristics, so it may not capture rapid changes in the body, which might be better noticed by the youngsters themselves when using the PDS^17^. Moreover, the PDS contemplates important changes related to puberty that the Tanner scale does not: growth in height^3^ and skin changes in both sexes^14^, menarche^1^ in females and both facial hair^13^ and voice changes^11^ in males. Additionally, the PDS can be particularly useful in assessing self-perception of young people when body image and self-esteem are of interest as well^2^.

Indeed, when scores of each PDS question were entered as separate levels in a PDS factor in GLM, we found that the variance explained in the PDS by Tanner ratings (R^2^) for each question were of medium to high effect sizes, with only one exception (R^2^ of Tanner pubic hair rating on the PDS breast growth question was 0.06, but that was still highly significant). Self-assessment of body hair growth was best related with pubic hair Tanner staging for both sexes (R^2^ = 53% males, 46% females), which makes sense as both are a consequence primarily of increases in adrenal hormones^9^^,^^14^. Growth spurt was related to pubic hair and breast/genital clinical staging in both sexes to a similar extent, confirming that it depends on both gonadal and adrenal stimulation^3^. In turn, for clinical staging of genital development in males, the most sensitive question was changes in voice (R^2^ = 51%), corroborating findings that this characteristic reflects gonadal effects^11^. Menarche explained 65% and 52% of breast and pubic hair clinical ratings respectively, showing that it relates more to gonadal than adrenal events, as it should^1^. Additionally, breast growth in the PDS was more associated with Tanner breast staging than pubic hair, which reflects the fact that breasts develop mostly because of gonadal stimulation^12^. Effect sizes in girls were smaller overall, probably because they were more sexually mature. Facial hair was the least sensitive question, unsurprisingly as it continues to grow well after Tanner stage 5^13^. Similarly, skin changes continue until the end of adolescence^14^.

Regarding the CFA, we found that for males all questions loaded onto a single factor and fit the data well. Differently, an adjustment had to be made in the model with female data to reflect the multidimensional (gonadal and adrenal) nature of the scale^1,8,11–14^. For both sexes, the excellent factor solutions and correlations of the pubertal factor with clinical ratings show that working in the CFA environment is a good alternative when using the PDS. This CFA approach to analyze PDS data is a novel finding in the literature.

Internal consistency of data from male and female answers in the PDS computed based on CFA results showed the reliability of the scale. Importantly, in the female model we found different magnitudes of factor loadings (ranging from 0.514 to 0.880). In such a situation, using Cronbach’s alpha is not recommend because the residual variances are correlated and the items do not load uniformly highly on the common PDS latent factor^25^^,^^27^. Although menarche is an unambiguous late event in gonadarche^2^, unlike the other PDS questions that have progressive ratings, menarche is a dichotomous variable and may impose limitations for Cronbach’s alpha calculation in our study and prior publications. Regardless of this, the reliabilities we found are similar to those of many other studies, which varied regarding higher alphas in males or females: this was so in the original version in English^9^^,^^10^ and the versions of the PDS translated into other languages such as Chinese^19^, Dutch^30^, Norwegian^31^, Spanish^32^, Finnish^33^, French^34^, and German^35^. In many cases, these alphas did not reach the lower acceptable boundary of 0.70^9^^,^^10^^,^^19^^,^^29^, while both our reliability measures did so (0.71 for females; 0.87 for males).

In the study described here, physicians who evaluated the participants were experts in the field and showed equivalent ratings until the middle of the experiment, so we did not assess inter-rater variability. However, we cannot exclude that some variability among raters may have occurred thenceforth. This could have potentially biased results. Additionally, note that determining inter-rater reliability of clinical staging is not the norm in studies that compared PDS to clinical pubertal staging. Therefore, in this respect, our study is comparable to those in the literature.

In sum, good estimates of pubertal development may be obtained from the adapted version of the PDS into Portuguese and are acceptable if exact correspondence to Tanner stages is not necessary. Still, our results should be replicated in larger clinical and non-clinical populations, ideally with more individuals in each pubertal stage than those in our sample. Nonetheless, we believe that our results make an important contribution to the literature by providing a Portuguese version of the PDS, showing factor structure of the scale, which has not been done previously, and evidencing, with a variety of statistical approaches, that PDS scores relate well, albeit not exactly, to clinical Tanner staging, confirming data in the few international publications that compared these metrics. We conclude that the Portuguese version of the PDS is adequate for determining pubertal staging when clinical ratings are not possible. This scale can, therefore, contribute to the understanding of the body changes that occur in adolescence.

## References

[1] 1. Grumbach MM. The neuroendocrinology of human puberty revisited. Hormone Res Paediatr. 2002;57 Suppl 2:2-14. 10.1159/000058094 12065920

[2] 2. Dorn LD, Dahl RE, Woodward HR, Biro F. Defining the boundaries of early adolescence: a user’s guide to assessing pubertal status and pubertal timing in research with adolescents. Appl Dev Sci. 2006;10(1):30-56. 10.1207/s1532480xads1001_3

[3] 3. Rogol AD, Roemmich JN, Clark PA. Growth at puberty. J Adolesc Health. 2002;31(6 Suppl):192-200. 10.1016/S1054-139X(02)00485-8 12470915

[4] 4. Marceau K, Ram N, Houts RM, Grimm KJ, Susman EJ. Individual differences in boys’ and girls’ timing and tempo of puberty: modeling development with nonlinear growth models*.* Dev Psychol. 2011;47(5):1389-409. 10.1037/a0023838 PMC3928626 21639623

[5] 5. Rockett JC, Lynch CD, Buck GM. Biomarkers for assessing reproductive development and health. Part 1- Pubertal development. Environ Health Perspect. 2004;112(1):105-12. 10.1289/ehp.6265 PMC1241804 14698938

[6] 6. Marshall WA, Tanner JM. Variations in pattern of pubertal changes in girls. Arch Dis Child. 1969;44(235):291-303. 10.1136/adc.44.235.291 PMC2020314 5785179

[7] 7. Marshall WA, Tanner JM. Variations in the pattern of pubertal changes in boys. Arch Dis Child. 1970;45(239):13-23. 10.1136/adc.45.239.13 PMC2020414 5440182

[8] 8. Rege J, Rainey WE. The steroid metabolome of adrenarche. J Endocrinol. 2012;214(2):133-43. 10.1530/JOE-12-0183 PMC4041616 22715193

[9] 9. Petersen AC, Crockett L, Richards M, Boxer A. A self-report measure of pubertal status: reliability, validity, and initial norms. J Youth Adolesc. 1988;17(2):117-33. 10.1007/BF01537962 24277579

[10] 10. Carskadon MA, Acebo C. A self-administered rating scale for pubertal development. J Adolesc Health. 1993;14(3):190-5. 10.1016/1054-139X(93)90004-9 8323929

[11] 11. Akcam T, Bolu E, Merati AL, Durmus C, Gerek M, Ozkaptan Y. Voice changes after androgen therapy for hypogonadotrophic hypogonadism. Laryngoscope. 2004;114(9):1587-91. 10.1097/00005537-200409000-00016 15475787

[12] 12. Berg SM, Setiawan A, Bartels M, Polderman TJ, Vaart AW, Boomsma DI. Individual differences in puberty onset in girls: Bayesian estimation of heritabilities and genetic correlations. Behav Genet. 2006;36(2):261-70. 10.1007/s10519-005-9022-y 16408250

[13] 13. Randall VA. Androgens and hair growth. Dermatol Ther. 2008;21(5):314-28. 10.1111/j.1529-8019.2008.00214.x 18844710

[14] 14. Ceruti JM, Leirós GJ, Balañá ME. Androgens and androgen receptor action in skin and hair follicles. Mol Cell Endocrinol. 2018;465:122-33. 10.1016/j.mce.2017.09.009 28912032

[15] 15. Mustanski BS, Viken RJ, Kaprio J, Pulkkinen L, Rose RJ. Genetic and environmental influences on pubertal development: longitudinal data from Finnish twins at ages 11 and 14. Dev Psychol. 2004;40(6):1188-98. 10.1037/0012-1649.40.6.1188 15535766

[16] 16. Schmitz KE, Hovell MF, Nichols JF, Irvin VL, Keating K, Simon GM, et al. A validation study of early adolescents’ pubertal self-assessments. J Early Adolesc. 2004;24(4):357-84. 10.1177/0272431604268531

[17] 17. Shirtcliff EA, Dahl RE, Pollak SD. Pubertal development: correspondence between hormonal and physical development. Child Dev. 2009;80(2):327-37. 10.1111/j.1467-8624.2009.01263.x PMC2727719 19466995

[18] 18. Hibberd EE, Hackney AC, Lane AR, Myers JB. Assessing biological maturity: chronological age and the pubertal development scale predict free testosterone in adolescent males. J Pediatr Endocrinol Metab. 2015;28(3-4):381-6. 10.1515/jpem-2014-0187 25332291

[19] 19. Chan NP, Sung RY, Nelson EAS, So HK, Yee KT, Kong AP. Measurement of pubertal status with a Chinese self-report pubertal development scale. Matern Child Health J. 2010;14(3):466-73. 10.1007/s10995-009-0481-2 19517073

[20] 20. Sadeh A, Dahl RE, Shahar G, Rosenblat-Stein S. Sleep and the transition to adolescence: a longitudinal study. Sleep. 2009;32(12):1602-9. 10.1093/sleep/32.12.1602 PMC2786044 20041596

[21] 21. Koo TK, Li MY. A guideline of selecting and reporting intraclass correlation coefficients for reliability research. J Chiropr Med. 2016;15(2):155-63. 10.1016/j.jcm.2016.02.012 PMC4913118 27330520

[22] 22. Landis JR, Koch GG. The measurement of observer agreement for categorical data. Biometrics. 1977;33(1):159-74. 843571

[23] 23. Ellis PD. The essential guide to effect sizes: statistical power, meta-analysis, and the interpretation of research results. New York: Cambridge University Press; 2010.

[24] 24. Muthén LK, Muthén BO. Mplus: the comprehensive modelling program for applied researchers: user’s guide, Los Angeles, CA: Muthén & Muthén; 1998-2017.

[25] 25. Raykov T. Scale reliability, Cronbach’s Coefficient Alpha, and violations of essential tau-equivalence with fixed congeneric components. Multivariate Behav Res. 1997;32(4):329-53. 10.1207/s15327906mbr3204_2 26777071

[26] 26. Dillon WR, Goldstein M. Multivariate analysis: methods and applications. New York: John Wiley; 1984.

[27] 27. Raykov T. Behavioral scale reliability and measurement invariance evaluation using latent variable modeling. Behav Ther. 2004;35(2):299-331. 10.1016/S0005-7894(04)80041-8

[28] 28. Hu L, Bentler PM. Cutoff criteria for fit indexes in covariance structure analysis: conventional criteria versus new alternatives. Struct Equ Modeling. 1999;6(1):1-55. 10.1080/10705519909540118

[29] 29. Brooks-Gunn J, Warren MP, Rosso J, Gargiulo J. Validity of self-report measures of girls’ pubertal status. Child Dev. 1987;58(3):829-41. 10.2307/1130220 3608653

[30] 30. Deković M, Noom MJ, Meeus W. Expectations regarding development during adolescence: parental and adolescent perceptions. J Youth Adolesc. 1997;26(3):253-72. 10.1007/s10964-005-0001-7

[31] 31. Wichstrøm L. The emergence of gender difference in depressed mood during adolescence: the role of intensified gender socialization. Dev Psychol. 1999;35(1):232-45. 10.1037/0012-1649.35.1.232 9923478

[32] 32. Siegel JM, Yancey AK, Aneshensel CS, Schuler R. Body image, perceived pubertal timing, and adolescent mental health. J Adolesc Health. 1999;25(2):155-65. 10.1016/S1054-139X(98)00160-8 10447043

[33] 33. Dick DM, Rose RJ, Pulkkinen L, Kaprio J. Measuring puberty and understanding its impact: a longitudinal study of adolescent twins. J Youth Adolesc. 2001;30(4):385-99. 10.1023/A:1010471015102

[34] 34. Verlaan P, Cantin S, Boivin M. [The scale of pubertal development: validation of the the French-language version of the Pubertal Development Scale]. Can J Behav Sci. 2001;33(3):143-7. French 10.1037/h0087136

[35] 35. Randler C, Bilger S, Díaz-Morales JF. Associations among sleep, chronotype, parental monitoring, and pubertal development among German adolescents. J Psychol. 2009;143(5):509-20. 10.3200/JRL.143.5.509-520 19943401

